# Supplementary material for: The salty tango of brine composition and UV photochemistry effects on Halobacterium salinarum cell envelope biosignature preservation
Source: Commun Biol. 2025 Apr 11;8:602. doi: 10.1038/s42003-025-08007-w (PMC11992018; doi:10.1038/s42003-025-08007-w)
Supplement: Supplementary file 2 — Supplementary Information [file 42003_2025_8007_MOESM2_ESM.pdf]

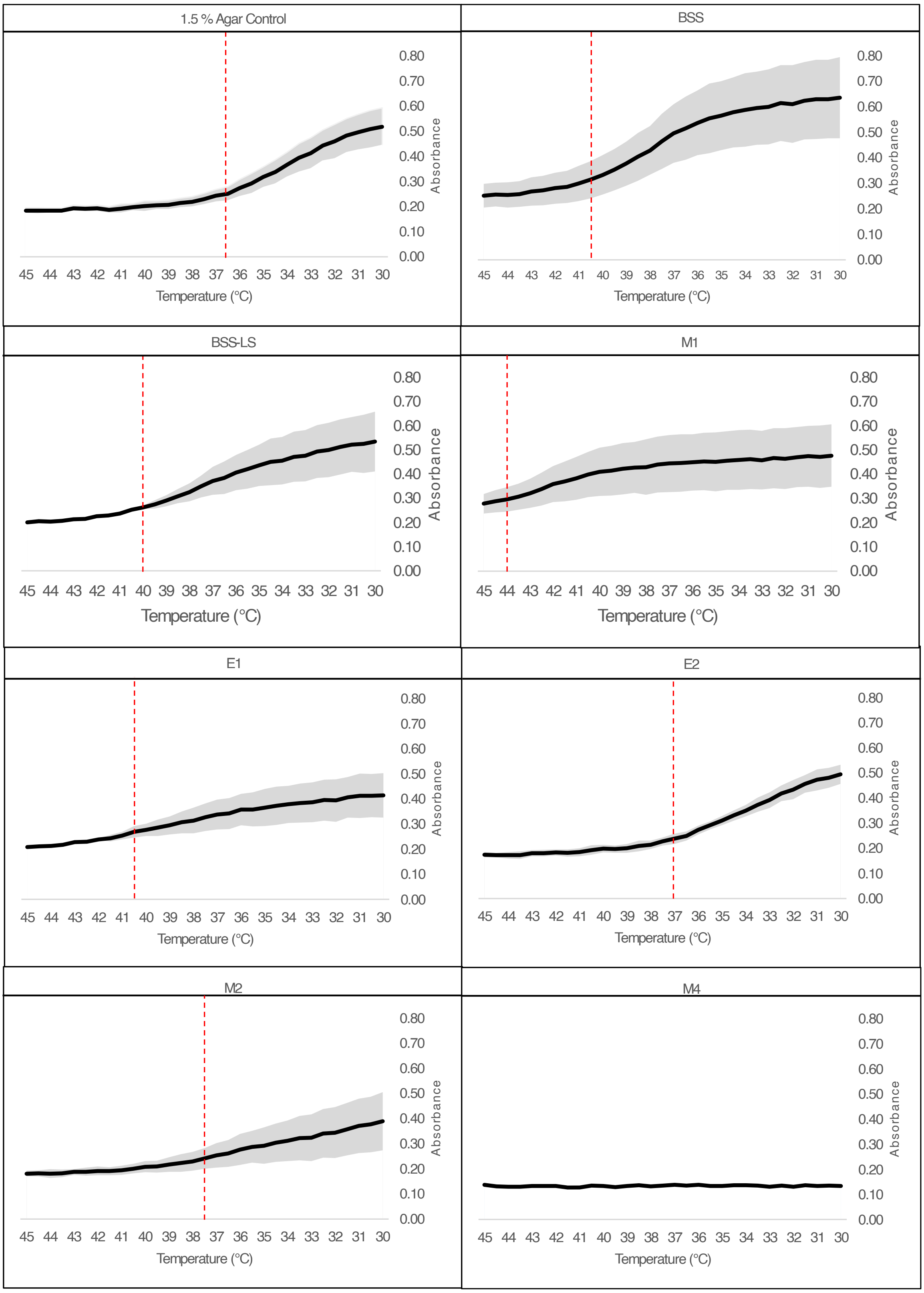

Supplementary Figure 1: Agar gel-point assay – Microplate method. Absorbance was measured at 500 nm over a temperature gradient from 45 °C to 30 °C at a 0.5 °C step. Each temperature decrease was followed by 5 min of incubation for equilibration. The colour of the iron-rich M3 brine was found to be incompatible with the microplate assay due to both the high absorbance of this solution at 500 nm and the high chaotropicity of the brine, which did not result in agar gelation under the temperature range used.

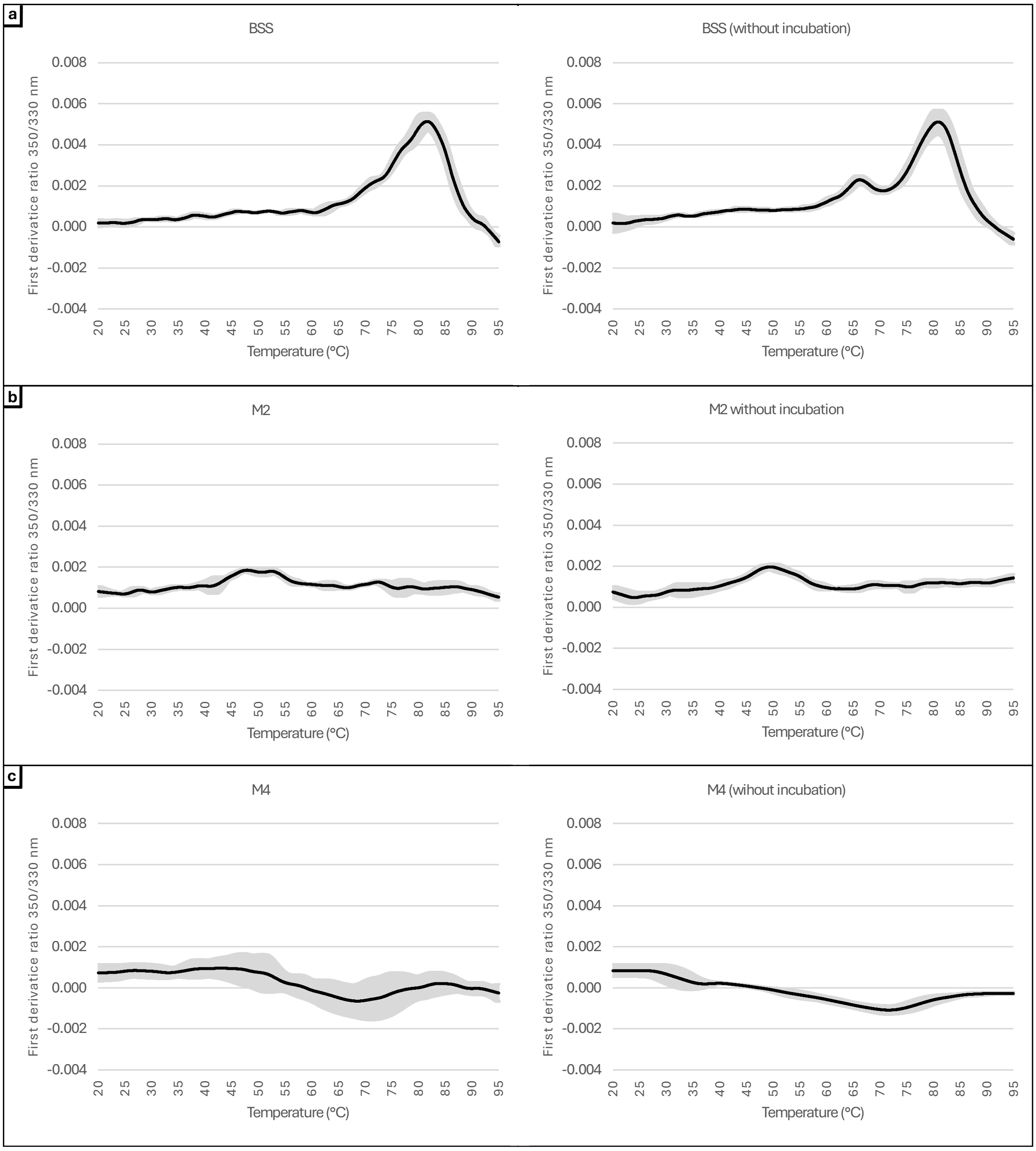

Supplementary Figure 2: NanoDSF curve of cell envelope extract pre-incubated or immediately resuspended ('without incubation') in brine solutions BSS, M1, and M4 (panels a-c, respectively) followed by NanoDSF analysis.

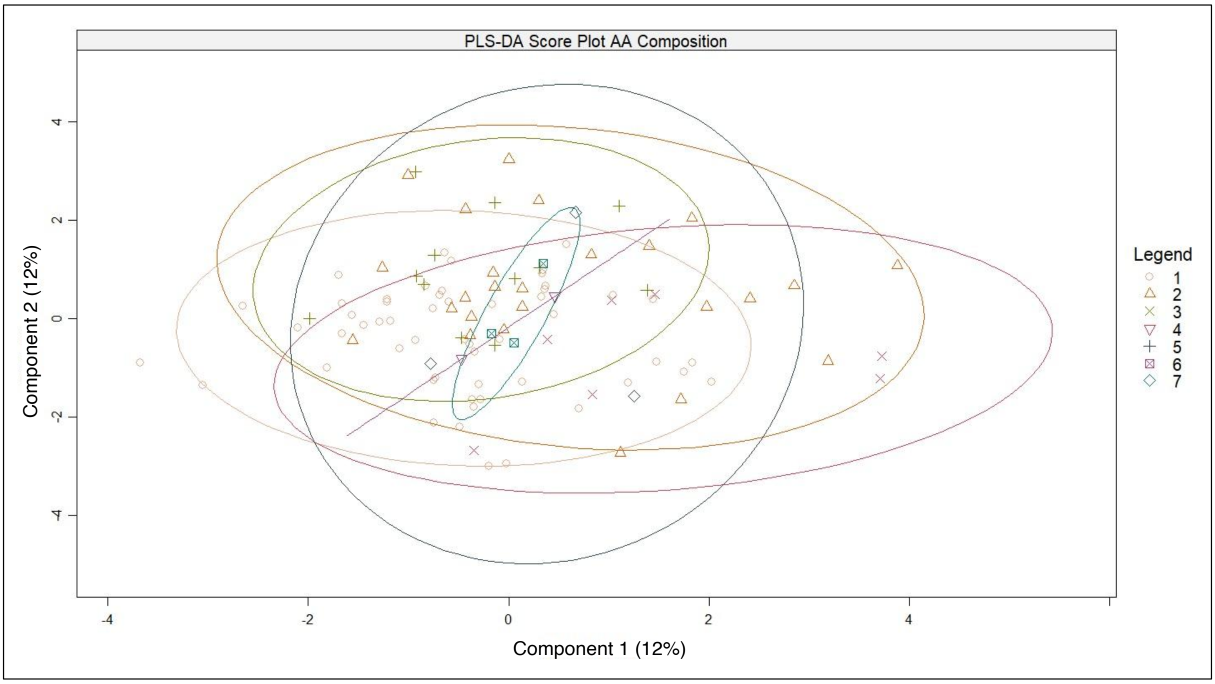

Supplementary Figure 3: PLS-DA of amino acids (AA) composition of the 106 proteins degraded by UV irradiation after incubation the different brines. All 106 proteins were analysed based on their amino acid composition and the numbers of brines within each protein was found to be degraded after incubation and UV irradiation (1-7, indicated by the respective symbols). Each symbol point corresponds to one protein with its specific amino acid composition. The total variance of the data was separated into Components 1 and 2, with the percentages representing the proportion of the total variance of the data explained by each component. Each circle indicates a group of proteins that were degraded in the same number of brines after UV exposure. No distinct separation of the groups was observed, indicating a lack of correlation between amino acid composition and sensitivity to UV irradiation.

Supplementary Table 1 : List of protein modifications identified as related to UV irradiation or reaction with specific reactive species or radicals (adapted from [1-3])

Amino Acid	Mass Shift
W,M,Y,F,H,L,I,R,V,T,P,K,C	15.9949
L,I,R,V,P,K,W,H	13.9793
M,W,Y,F,C,H	31.9898
M	-31.9898
C	-15.9772
C,W,H	47.9847
H	-23.016
H	-22.032
H	-10.032
R	-43.0534
D,E	-30.0106
M	-50.019
W	3.9949
M	-32.0085
W	19.9898
W	-59.0524
W	-71.0524
W,H	29.9742
W	-43.0575
H	-4.0313
H	4.9789
H	50.0004
C	-31.9721
C	-33.9877
C	42.0106
Y	44.9851
Y	33.961028

→

Amino Acid	Mass Shift	Composition change
W,M,Y,F,H,L,I,R,V,T,P,K,C	15.994915	+ O
M,W,Y,F,C,H	31.989829	+ 2O
W	3.994915	+O, -C
L,I,R,V,P,K,W,H	13.979265	+ O, - 2H
D,E	-30.010565	-C, - 2H, - O
H	-22.0326	$+O_2, -C_2H_2N_2$
M	-32.008456	+ O, - S, - C, - 4H
H	-10.031969	+ 2O, - C, - 2N, - 2H
H	-23.015	$+O_2, -C_2HNO$
Y	33.961028	+Cl
C,W,H	47.984744	+ 3O
W	19.989829	$+O_2, -C$
C	42.010565	$+CH_2CO$
Y	44.985078	$+NO_2$

Supplementary Table 2: List of proteins identified by mass spectrometry only in the control *Halobacterium salinarum* cells cultured either under control conditions (BSS) or cells acclimated to growth in the E1 brine.

	Only identified in control condition (BSS)
Protein Entry	Protein Name
Q9HHV6	Vng6208c
Q9HQ54	Pectin lyase domain protein
Q9HRN6	Htr16 transducer
Q9HT02	ABC transporter permease
Q9HRC9	Glutamate/valine-rich protein
Q9HSA0	DUF3007 family protein
Q9HHN9	Vng6302c
Q9HNG6	Mechanosensitive channel protein MscS
Q9HNP0	M50 family metalloprotease (EC 3.4.24.-)
Q9HPT0	Uncharacterized protein
Q9HNI4	Uncharacterized protein
Q9HQP3	Succinoglycan biosynthesis protein
Q9HR88	Htr18 transducer
Q9HPX5	AI-2E family transport protein (Htr-like protein)
Q9HS82	Rhomboid family protease (EC 3.4.21.105)
Q9HR23	ABC-type transport system permease protein(Probable substrate sugar) (Ribose ABC transporter permease)
Q9HMI2	ABC-type transport system permease protein(Probable substrate dipeptide/oligopeptide) (Dipeptide ABC transporter permease)
Q9HRH6	ABC-type transport system permease protein
Q9HS33	Major facilitator superfamily transport protein (Multidrug resistance protein homolog)
Q9HQ30	DUF304 domain protein
Q9HRH7	ABC-type transport system permease protein
O51960	Vng6008h
ARCD	Arginine/ornithine antiporter ArcD
Q9HPA2	Htr17 transducer (Transducer protein Htr17)
Q9HQQ6	Dolichyl-phosphate glucosyltransferase AglJ (EC 2.4.1.117) (Glycosyl transferaselike)
Q9HMW3	ABC-type transport system permease protein(Probable substrate dipeptide/oligopeptide) (Oligopeptide ABC permease)
Q9HRH2	Molybdenum-binding protein (P-type transport ATPase (Probable substratecopper/metal cation) (EC 3.6.3.-))
Q9HQH2	Mechanosensitive channel protein MscS
	Only Identified in acclimated sample (E1)
Q9HPW7	Uncharacterized protein
Q9HHS5	DUF2078 family protein (Vng6251h)
Q9HMK0	Uncharacterized protein
BLH	Probable beta-carotene 15,15'-dioxygenase Blh (EC 1.13.11.63) (Brp-like homolog)
Q9HMC9	Arsenite transport protein (DASS family transport protein)
Q9HRL2	F420H2:quinone oxidoreductase chain M (NADH dehydrogenase-like complex subunit M)
BACR	Bacteriorhodopsin (BR) (Bacterioopsin) (BO)
Q9HS59	DUF373 family protein
O52005	YidE family protein
Q9HNI9	ABC-type transport system permease protein(Probable substrate phosphate/phosphonate) (Transport protein)
Q9HP95	ZIP family metal transporter
Q9HPA6	DUF106 family protein (Htr-like protein)
Q9HSA7	NfeD domain protein
Q9HNF6	Probable NhaC-type sodium/proton antiporter
Q9HMX0	ABC-type transport system permease protein(Probable substrate dipeptide/oligopeptide) (Dipeptide ABC transporter permease)
Q9HRT4	DUF2298 family protein
Q9HSL5	Cationic amino acid transporter

Supplementary Table 3: Overview of the different reactive species and radicals being produced from specific molecules or ions under UV-C irradiation.

Ions in solutions	Reactive Species/Radicals generated	References
**H_20**	$H_20^{\bullet}$, $^{\bullet}OH$, $O_2^{\bullet -}$	[4,5]
**Cl^-**	Cl_2^{-}, Cl_3^{-}, $Cl_2^{-\bullet}$, $^{\bullet}Cl$, $ClOH^{\bullet -}$, $^{\bullet}OCl$, $HOCl/OCl^-$	[6]
**SO_4^{2-} -**	$SO_4^{\bullet -}$, HSO_4^{-}	[7,8]
**HCO_3^-**	$CO_3^{\bullet -}$, $HCO_3^{\bullet}$	[3,9]
**ClO_4^-**	ClO_3^{-}, ClO_2^{-}, ClO^-, Cl^-, $ClO_2^{\bullet}$, $^{\bullet}OH$, $O^{\bullet -}$	[10]

UV	Irradiance (J/s/m^2)
UVA (400-320 nm)	33.5
UVB (320-280 nm)	29.5
UVC (280-100 nm)	93.8
Total	156.8

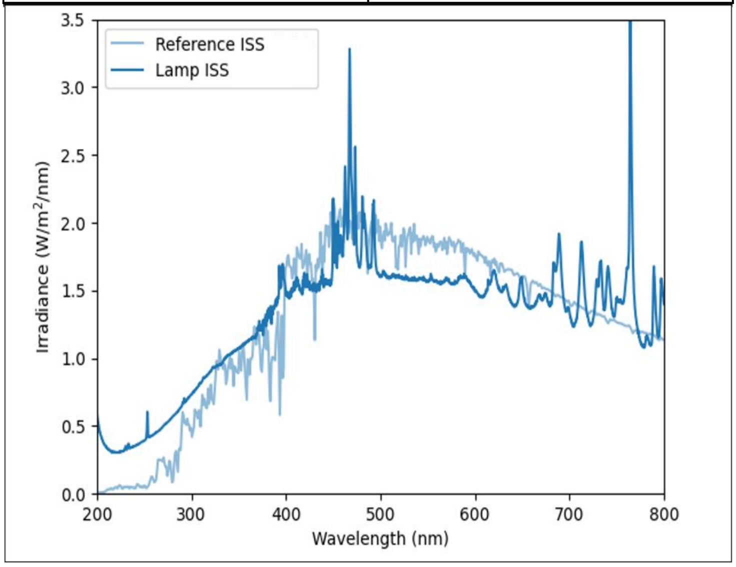

Supplementary Figure 4 : Solar simulator irradiance recorded using Xe lamp (Hamamatsu L2479). “Reference ISS” corresponds to the reference spectrum of the irradiance in the orbit of the International Space Station and “Lamp ISS” corresponds to the spectrum recorded from the Xe lamp used in this study.

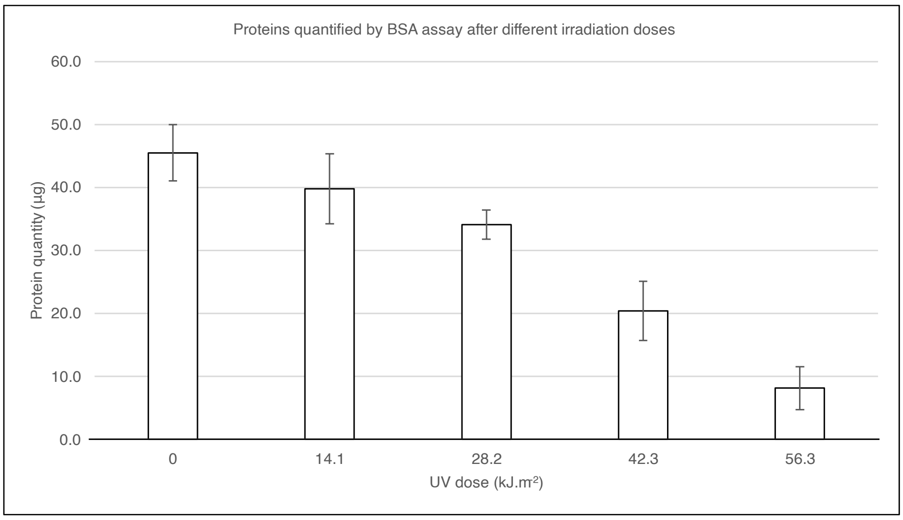

Supplementary Figure 5 : Membrane protein quantification by BCA after different exposure time to UV radiation. Irradiation dose exceeding 14.1 kJ.m^{-2} induced significant protein loss, while irradiations dose more than 42.3 kJ.m^{-2} resulted in insufficient protein recovery for downstream analyses. Therefore, a dose of 28.2 J k.m^{-2} was chosen as standard irradiation exposure time for all subsequent irradiations.

Supplementary Table 4: Composition of the media used in the stepwise approach for the acclimation experiment.

	E1	CONTROL CM medium				
For 100ml (g)	End Point	Start Point	Acclimation Step #1	Acclimation Step #2	Acclimation Step #3	Acclimation Step #4
KCl	2.01	0.2	0.653	1.105	1.558	2.01
$MgCl_2.6H_2O$	3.66	0	0.915	1.83	2.745	3.66
NaCl	32.49	25	26.873	28.745	30.618	32.49
$MgSO_4.7H_2O$	0	2	1.5	1	0.5	0
Na_2SO_4	1.14	0	0.285	0.57	0.855	1.14
Trisodium citrate.$2H_2O$	0	0.3	0.225	0.15	0.075	0
Peptone (Oxoid®)NON-NEUTRALIZED	1	1	1	1	1	1

	E2	CONTROL CM medium				
For 100ml (g)	End Point	Start Point	Acclimation Step #1	Acclimation Step #2	Acclimation Step #3	Acclimation Step #4
KCl	3.95	0.2	1.1375	2.075	3.0125	3.95
$MgCl_2.6H_20$	27.04	0	6.76	13.52	20.28	27.04
NaCl	15.55	25	22.6375	20.275	17.9125	15.55
$MgSO_4.7H_20$	0	2	1.5	1	0.5	0
$CaCl_2$	8.82	0	2.205	4.41	6.615	8.82
Trisodium citrate.$2H_2O$	0	0.3	0.225	0.15	0.075	0
Peptone (Oxoid®)NON-NEUTRALIZED	1	1	1	1	1	1

	M2	CONTROL CM Medium				
For 100ml (g)	End Point	Start Point	Acclimation Step #1	Acclimation Step #2	Acclimation Step #3	Acclimation Step #4
KCl	7.7	0.2	2.075	3.95	5.825	7.7
$MgCl_2.6H_2O$	23.46	0	5.865	11.73	17.595	23.46
NaCl	13.24	25	22.06	19.12	16.18	13.24
$MgSO_4.7H_2O$	62.85	2	17.2125	32.425	47.6375	62.85
HCl	0.14	0	0.035	0.07	0.105	0.14
Trisodium citrate. H_2O	0	0.3	0.225	0.15	0.075	0
Peptone (Oxoid®)NON-NEUTRALIZED	1	1	1	1	1	1

References

1. Wang, L. & Chance, M. R. Structural mass spectrometry of proteins using hydroxyl radical-based protein footprinting. *Anal. Chem.* **83**, 7234–7241 (2011).
2. Nybo, T., Davies, M. J. & Rogowska-Wrzesinska, A. Analysis of protein chlorination by mass spectrometry. *Redox Biol.* **26**, (2019).
3. Li, J. et al. Hydrogen peroxide formation in water during the VUV/UV irradiation process: Impacts and mechanisms of selected anions. *Environ. Res.* **195**, (2021).
4. Piskarev, I. M. Hydrogen peroxide formation in aqueous solutions under UV-C radiation. *High Energy Chem.* **52**, 212–216 (2018).
5. Piskarev, I. M., Astaf'Eva, K. A. & Ivanova, I. P. The UV-C radiation mechanism to form HO_2• radicals from water. in *J. Phys.: Conf. Ser.* **1094**, (Institute of Physics Publishing, 2018).
6. Wu, D., Wong, D. & Di Bartolo, B. Evolution of Cl^{-}_2 in aqueous NaCl solutions. *J. Photochem.* **14**, 303–310 (1980).
7. Barrett, J., Fox, M. F., Mansell, A. L. & Barrett, J. The photochemistry of aqueous sulfate ion. *J. Phys. Chem.* **69**, 723–730 (1965).
8. Vu, X. Y., Bao, Z. C. & Barker, J. R. Free radical reactions involving Cl•, Cl_2^{-}•, and SO_4^{-}• in the 248 nm photolysis of aqueous solutions containing $S_2O_8^{2-}$ and Cl^{-}. *J. Phys. Chem. A* **108**, 295–308 (2004)
9. Chen, Y. et al. Effect of magnesium on reducing the UV-induced oxidative damage in marrow mesenchymal stem cells. *J. Biomed. Mater. Res. A* **107**, 1253–1263 (2019).
10. Georgiou, C. D., Zisimopoulos, D., Kalaitzopoulou, E. & Quinn, R. C. Radiation-driven formation of reactive oxygen species in oxychlorine-containing Mars surface analogues. *Astrobiology* **17**, 319–336 (2017).
