# Supplementary material for: The salty tango of brine composition and UV photochemistry effects on Halobacterium salinarum cell envelope biosignature preservation
Source: Commun Biol. 2025 Apr 11;8:602. doi: 10.1038/s42003-025-08007-w (PMC11992018; doi:10.1038/s42003-025-08007-w)
Supplement: Supplementary file 3 — Description of Additional Supplementary Files [file 42003_2025_8007_MOESM3_ESM.pdf]

Description of Additional Supplementary Files

File: Supplementary Data 1
Description: The source data for graphs in the paper

File: Supplementary Data 2
Description: The source data for graphs in the paper

File: Supplementary Data 3
Description: The source data for graphs in the paper
